# Reducing veterans’ risk for suicidal behaviors: a qualitative study to inform development of the RECLAIM health promotion program

**DOI:** 10.1186/s12913-020-05587-7

**Published:** 2020-08-01

**Authors:** Sarah Shue, Jayme Brosmer, Marianne S. Matthias

**Affiliations:** 1 VA HSR&D Center for Health Information and Communication, Roudebush VA Medical Center, 1481 West 10th Street, Indianapolis, IN 46202 USA; 2 VA Physical Medicine and Rehabilitation Service, Roudebush VA Medical Center, Indianapolis, IN USA; 3 Regenstrief Institute, Indianapolis, IN USA; 4 Department of Medicine, Indiana University School of Medicine, Indianapolis, IN USA

**Keywords:** Military, Post-9/11, Clinicians, Focus groups, Community-based, Holistic

## Abstract

**Background:**

In an effort to reduce the high rate of suicide among post-9/11 veterans, a collaborative team within the Department of Veterans Affairs (VA) has developed a holistic community-based health promotion program designed to facilitate social and self-connectedness. The purpose of this study was to elicit veteran and stakeholder feedback to prepare the program for piloting and implementation.

**Methods:**

Focus groups and interviews were conducted with post-9/11 veterans and veteran stakeholders (e.g., VA clinicians) to elicit feedback regarding the health promotion program. Focus groups and interviews were audio-recorded and transcribed. Qualitative thematic analysis identified key themes emerging from the focus groups and interviews.

**Results:**

Seven focus groups (3 Veteran groups, 4 stakeholder groups) and 3 interviews (2 Veterans, 1 stakeholder) were conducted with 41 participants (14 veterans, 27 stakeholders). Overall, participants had a positive perception of the program. Thematic analysis revealed shared perspectives that provided insight into 1) enhancing program recruitment and retention, 2) the perceived ability of a health promotion program to provide more holistic, veteran-centered care, and 3) using health promotion programs to help veterans establish structure in their daily lives.

**Conclusions:**

Findings indicated an overall acceptance of the program, and participants’ perspectives on how to reduce barriers and enhance facilitators can inform the development of a larger-scale health promotion program that can be tested through future research. While discussion questions were specifically focused on the program in this study, findings can be considered more broadly for the design and implementation of related programs to effectively improve the health and wellness of post-9/11 veterans.

## Background

Veterans frequently face a sense of disconnectedness in their social lives (i.e., loneliness, social isolation) and personal lives (i.e., self-connectedness – lack of purpose or goals) when they leave the military [1–4]. This lack of connectedness, both social and self, has been established as a risk factor for suicidal behaviors [5]. However, few interventions, especially suicide prevention efforts, seek to improve veterans’ overall connectedness or focus on the reintegration experience specifically.

While efforts have been made to prevent and reduce veteran suicide, the rate of suicide among post-9/11 veterans (ages 18–34) remains the highest of all veteran cohorts and has increased by 76% from 2005 to 2017 [6], indicating that more work is needed to effectively reduce the rate of suicide among this veteran cohort. Current suicide reduction and prevention strategies often narrowly focus on extreme psychopathology (e.g., post-traumatic stress disorder) and are frequently directed at veterans who have already indicated intent to harm [7, 8]. However, many veterans not reporting physical or psychological complaints still indicate difficulties in social functioning (i.e., social connectedness), self-care (i.e., self-connectedness), and other major life domains, which all can lead to harmful behaviors [9]. Therefore current strategies may overlook veterans for whom suicide prevention efforts could be helpful. Recognizing the potential limitations of current approaches, the Department of Veterans Affairs (VA) issued a National Strategy for Preventing Veteran Suicide, which suggests that “while the focus of veteran suicide prevention is predominantly on counteracting risk factors, strengthening protective factors can help prevent suicide by promoting physical, mental, emotional, and spiritual wellness” [10] (p.17). Research has demonstrated that strengthening protective factors and improving psychological well-being (i.e., positive relationships, autonomy, purpose, personal growth) can protect from harmful, suicidal behaviors [11, 12]. However, a limited number of interventions purposefully target psychological well-being to reduce the risk of suicidal behaviors [13].

### Theoretical background

The interpersonal psychological theory of suicide (IPT) is a prominent theory of suicidal thoughts and behaviors [5]. The IPT suggests that suicidal ideation is comprised of two interpersonal constructs: thwarted belongingness (e.g., feeling isolated, not belonging to a group) and perceived burdensomeness (e.g., belief that one’s existence places an extreme burden on other people). The IPT proposes that even if suicidal ideation is present, any attempt (lethal or non-lethal) is unlikely unless the individual also possesses the capability for suicide. However, while the IPT identifies pathways for suicidal behaviors, it does not guide how to reduce the risk of suicidal behaviors and improve psychological well-being; therefore, a second theory was identified to support development of a program focused on improving well-being, which can help reduce the risk of suicidal behaviors. The constructs of the IPT align with a theory of self-regulation and motivation known as the self-determination theory (SDT). The SDT focuses on three universal psychological needs: relatedness (belonging), competence (mastery), and autonomy (control) [14, 15]. These theories were used in this study to inform each other and expand our understanding of how to effectively address and improve protective factors among veterans to improve their social and self-connectedness and reduce their risk of suicidal behaviors.

In alignment with the recommendation from the VA to address protective factors and to address the gap in understanding how to strengthen protective factors to prevent suicidal behaviors, a collaborative team within the VA has developed a holistic health promotion program theoretically founded in the IPT and SDT. This program, known as RECLAIM (REconnecting to Civilian Life using Activities that Incorporate Mindfulness), focuses on the broad range of factors impacting veterans’ health and address the whole person (e.g., emotional, physical, spiritual, social wellness) to facilitate overall connectedness and reduce the risk of suicidal behaviors among post-9/11 veterans. As RECLAIM was being prepared for piloting, it was important that post-9/11 veterans, who may be future participants in the program, and veteran stakeholders, who would have the ability to recommend or refer veterans to the program, were involved in the design of the program. The purpose of this study was to elicit feedback from veterans and stakeholders regarding initial perceptions of the RECLAIM program and considerations for future implementation.

## Methods

### The RECLAIM program

RECLAIM is a community-based program that incorporates mindfulness strategies (i.e., imagery, yoga, meditation) and is theoretically grounded in the IPT and the SDT. RECLAIM is designed to facilitate veterans’ social and self-connectedness as they reintegrate back into civilian life and, ultimately, reduce veterans’ risk of engaging in suicidal behaviors. RECLAIM is comprised of 8 face-to-face group sessions and self-guided home assignments. Social connectedness will be facilitated through group discussion and recommended community resources and activities, while engaging and developing competence in mindfulness activities and self-guided work will enable veterans’ self-connectedness. The second author (JB), who is trained in mindfulness activities and techniques, will facilitate the program sessions.

RECLAIM will be conducted within the VA Wellness Clinic, which is located inside a Midwest YMCA (Young Men’s Christian Association) facility. This is currently the only VA clinic in the country to be housed within a YMCA. This clinic offers physical and operational therapy, pain management, and other counseling services. Majority of the focus groups and interviews were conducted in private offices within the VA Wellness Clinic. One focus group and one interview took place at the nearby VA medical center.

### Design

Focus groups were conducted to elicit veteran and stakeholder feedback on the RECLAIM program. Focus group questions were designed to encourage discussion of barriers and facilitators to participation, program design preferences (e.g., content, number and length of sessions), and suggestions for improvement. Participants interested in providing feedback but unable to participate in a focus group time slot were invited to engage in an individual interview using the same questions as the focus groups. This study’s procedures received exempt approval from the local institutional review board (IRB; Indiana University) and the Richard L Roudebush VA Medical Center’s Research and Development committee. Veterans and stakeholders verbally consented to study participation prior to beginning audio recording of discussions. To protect participant identity, each participant was assigned a participant ID, which indicated whether the participant was a veteran or a stakeholder.

### Selection of participants

We used purposive sampling (snowballing and maximum variation strategies) to recruit veteran and stakeholder participants [16]. For veterans to be eligible for participation, they must have served after October 2001 and be within 10 years of exiting their military service. Stakeholders were either VA or YMCA employees. Considering the RECLAIM program will be conducted at a unique community-based clinic within a YMCA, it was important to include YMCA employees’ perspectives, as they could refer their veteran patrons to the program. Eight focus groups (four veteran, four stakeholder) were scheduled and each was designed to accommodate 10 participants [17]. Participant recruitment ceased when the sample size met published recommendations on qualitative sample size [17, 18] and thematic saturation occurred [19].

### Data collection

Focus groups and interviews took place in a private conference room in either the community-based VA Wellness Clinic at the YMCA or at the nearby VA medical center. Participants were given a study information sheet prior to conducting the focus group. Each participant completed a demographics form (age, gender, employment and marital status). Veteran participants also completed outcome measures (connectedness, mindfulness, personal health goals) to assess feasibility (i.e., time to complete) for future studies regarding the developing program.

Participants were provided a verbal overview of the planned program and a handout to facilitate discussion. Once participants verbally consented to participation, the audio recording of the discussion began. To achieve the objectives of this study, a semi-structured interview guide was developed using a grounded theory approach [20]. The interview guide questions are provided as Supplementary file 1. The interview guide questions were based on suggestions from previous research [18, 21] and aimed to elicit participants’ feedback regarding the most and least appealing aspects of RECLAIM, suggested changes, potential benefits, and barriers to participation. The same guide was used in the focus group discussions and the individual interviews. Additionally, the same guide was used for both veterans and stakeholders with the exception of the final question, which was worded to either understand a) what would make veterans want to participate (veteran groups/interviews) or b) what would make stakeholders recommend the program to veterans (stakeholder groups/interviews). The discussion was moderated by the lead investigator (SS) and assisted by one of the co-investigators (JB). Both investigators took notes during each group. Data collection ceased when thematic saturation occurred (i.e., no unique findings emerged from focus group discussions) [19].

### Data analysis

Focus groups were recorded, transcribed, and de-identified. Transcripts were reviewed while listening to the audio to ensure accuracy. All data were analyzed using inductive coding and a thematic analysis approach [19]. In alignment with recommended steps for conducting rigorous thematic analysis [22], the first and second authors (SS and JB) iteratively reviewed the data and field notes. Then, the first and second authors analyzed the transcripts independently to develop a preliminary set of codes. The independent interpretations and code sets developed by the two authors were compared and discussed until an agreement was reached and single set of codes was developed. As transcripts continued to be reviewed these codes were applied to each transcripts’ text to determine whether edits, such as adding, combining, or eliminating codes were needed. The transcripts were repeatedly read and reviewed by the first and second author to identify themes across all transcripts. Once themes were developed, the transcripts were revisited to ensure raw data were adequately incorporated and the third author (MM) reviewed the developed themes and transcript data to ensure agreement. A team consensus was reached on the theme names.

## Results

### Participants

Seven focus groups with 38 participants (12 veterans and 26 stakeholders) were conducted between August and October 2019. Focus groups lasted approximately 60 min. Three veteran focus groups were held and ranged from 2 to 5 participants. Four stakeholder focus groups were held and ranged from 4 to 10 participants. Three participants (two veterans and one stakeholder) were unable to make a focus group and, instead, engaged in a one-on-one interview. All interviews lasted approximately 30 min. This provided a total of 41 participants. Participants’ age ranged from 24 to 65 years old and the average age of all participants was 39.6 (SD = 10.1). Additional participant demographics are provided in Table 1.

**Table 1:** Veteran and stakeholder demographics

	All (*N* = 41)		Veterans (*n* = 14)		Stakeholders (*n* = 27)	
	N	%	N	%	N	%
**Gender**						
Female	26	63.4	6	42.8	20	74.1
Male	15	36.6	8	57.1	7	25.9
**Marital status**						
Married/Civil Union	26	63.4	6	42.9	20	74.1
Engaged or in a relationship	2	4.9	–	–	2	7.4
Single	8	19.5	4	28.6	4	14.8
Separated	1	2.4	1	7.1	–	–
Divorced	4	9.8	3	21.4	1	3.7
**Education**						
High school diploma/GED	1	2.4	1	7.1	–	–
Some college/2-year degree	3	7.3	1	7.1	2	7.4
4-year college graduate	15	36.6	5	35.7	10	37
More than a 4-year degree	22	53.7	7	50	15	55.6

#### Veteran participants

The average age of veteran participants was 38.5 (SD = 11.2). Most participants had served in the Army (71.4%) and had seen combat (78.6%). Most veterans indicated they were employed (64.3%), several were retired (28.6%), and a few were unable to work due to disability (21.4%). All veteran participants were receiving VA care at the time of study participation.

#### Stakeholder participants

The average age of stakeholder participants was 40.2 (SD = 9.7). Most stakeholder participants were YMCA employees (51.9%). Five stakeholder participants (18.5%) indicated being military veterans.

### Emergent themes

Analysis revealed three main themes: 1) enhancing program recruitment and retention, 2) the perceived ability of a health promotion program to provide more holistic, veteran-centered care, and 3) using health promotion programs to help veterans establish structure in their daily lives. These themes reflect the perspectives of both veterans and stakeholders. Significantly differing perspectives between veterans and stakeholders are highlighted within the theme discussions below. This section, in alignment with rigorous thematic analysis, provides detailed descriptions and quotes to aid the understanding of how the themes emerged [22]. The participant ID is provided after the identifier (i.e., veteran, VA stakeholder, YMCA stakeholder) in parenthesis. Table 2 provides an overview of the three themes and their alignment with the constructs of the two theories (IPT and SDT) that informed the development of the RECLAIM program.

**Table 2:** Theme overview

Theme	Sample statements	Theoretical constructs
**Theme 1: Enhancing program recruitment and retention** *Subthemes*: ▪ Being clear on purpose and process ▪ Creating a sense of familiarity (e.g., terminology, technology)	▪ “*The more that it feels like home and familiar, the more that [you create] that draw*.” – Veteran ▪ “*This is something that you should think about for people that are getting out, like your out-processing type of briefings*.” – Veteran	▪ Belonging (IPT) ▪ Relatedness (SDT)
**Theme 2: The perceived ability of a health promotion program to provide more holistic, veteran-centered care** *Subthemes:* ▪ Considering the whole person ▪ Proactive care ▪ Community-based setting versus typical health care facility	▪ “*If we can say we’re interested in actually understanding what you’re passionate about so that we can program and strategically plan as an organization toward those types of things, it’d just be helpful*.” – VA stakeholder	▪ Autonomy (SDT) ▪ Competence (SDT) ▪ Burdensomeness (IPT) ▪ Belonging (IPT) ▪ Relatedness (SDT)
**Theme 3: Using health promotion programs to help veterans establish structure in their daily lives** *Subthemes:* ▪ Establishing a mission/purpose ▪ Building a support system	*▪“We have to get past that barrier to get them to understand that they have to unlearn all this [military] stuff. It’s just like going through the military again. You’re re-learning how to live in a completely different way now.” –* YMCA stakeholder ▪ “*You change the way you think when you’re in and then you have to figure out how to change the way you think when you get out*.” – VA stakeholder/veteran	▪ Autonomy (SDT) ▪ Competence (SDT) ▪ Burdensomeness (IPT) ▪ Belonging (IPT) ▪ Relatedness (SDT)

*IPT* Interpersonal Psychological Theory of Suicide, *SDT* Self-Determination Theory

#### Enhancing program recruitment and retention

Focus group participants frequently discussed how to get veterans interested in a health promotion program, what would be necessary for buy-in, and how to keep veterans engaged in program activities, even after the program concludes.

##### Being clear on purpose and process

Participants discussed how veterans would be reluctant to participate if the program’s purpose and activities were vague. It was frequently suggested that peer-to-peer outreach (i.e., former program participants should be invited to speak about their experience) should be used to promote the program and create immediate buy-in.

“If you could have a [veteran] that [others] respected and could sit down with everybody, all of the guys that are getting out or whatever and you talk about these things and be like look, y’all are about to get out. The transition is difficult. Like if you have a respected [veteran] talk about this…the [veterans] would listen.” – Veteran (V12)

Participants with *previous* experience practicing mindfulness techniques suggested being clear on the level of difficulty involved in learning the proposed activities.

“These things are not very easy either, right? They are something that require discipline and daily practice. It took me some time to accept that in order to live a normal life again or anything resembling that, that it was going to take effort, constant effort every single day to get from where I was to where I wanted to be.” – Veteran (V2)

“I know how difficult it is to immerse yourself in [mindfulness] work and how brutal, and it requires a lot of like self-accountability and finding the right community support. I love this stuff, but I love being honest about the work that goes into this.” – YMCA stakeholder (S20)

Veteran participants also felt that some of the proposed terminology, such as ‘lifestyle program,’ was “*invasive*” and “*vague*” and the lack of clarity would not appeal to a broad veteran audience and could leave veterans wondering “*what [they] would share with you*.”

“I think that [lifestyle] is kind of a scary word. Because it’s kind of vague. Do you know what I mean? Like it’s a little vague. It might mean different things.” – Veteran (V7)C

Use of the term ‘lifestyle’ also made veteran participants question, “*why isn’t my lifestyle right now [legitimate]?*” or state that “*there is nothing wrong with my lifestyle*.”

##### Creating a sense of familiarity

Participants repeatedly suggested that RECLAIM promoters needed to find ways to make the program stand out considering veterans receive a lot of information when they exit the military. Veterans suggested “*piggybacking*” onto existing military or VA programs to “*prime*” service members for participation in RECLAIM. Participants also suggested reaching out to individuals before they exit the military to establish a sense of familiarity with RECLAIM prior to transitioning back into civilian life.

“…before people get out of [the military] introducing them to some of these things and programs that are paired up. [Letting them know that] the VA has something that’s similar so that when they get out, it looks and feels like home to them. Because the military was home.” – Veteran (V13)

Participants were also considerate of the fact that many veterans may not be in the frame of mind to consider this type of program upon exit from the service and “*may reject it immediately*” (Veteran, V3). Even with these challenges, most participants strongly recommended recruiting veterans in the early stage of their career transition.

“Transitioning out of the military, like this would have been perfect. Because, even us younger folks, like, I’m out and I’ve never done anything else. I don’t know how to be an adult.” – Veteran (V8)

“They send out a bunch of stuff, but it’s never anything that pertains to anybody especially like when you first get out of the military. So getting [RECLAIM] to those [veterans] is probably one of the bigger things.” – Veteran (V2)

However, some veterans talked about still needing this type of program even though they had transitioned out several years ago.

“I wish I would’ve had an opportunity like this when I came back from Afghanistan. I think it would’ve made a huge difference for me in my reintegration process. You know, I’ve been home for 7 years and it still feels like yesterday to me. I mean it literally feels like yesterday.” – Veteran (V1)

In addition to providing clarity, terminology was discussed by participants to create a sense of familiarity regarding RECLAIM and its concepts. Participants repeatedly suggested use of the term “resilience” because it is frequently used in the military.

“We always talked about it in the military, being able to build a resilient warrior, right? … Most people that have been around the military would be like, okay, I’m identifying and understanding that context.” – YMCA stakeholder/veteran (S2)

Participants encouraged the inclusion of technology, such as apps or podcasts, which would be familiar to younger “*tech savvy*” (Veteran, V12) veterans, enhance RECLAIM’s appeal, and increase the likelihood of recruitment and retention of these veterans. This would also allow veterans to access program-related independently, which would support long-term engagement. Apps where veterans could check in were frequently suggested for establishing a sense of accountability and improve program retention.

#### The perceived ability of a health promotion program to provide more holistic, veteran-centered care

Participants expressed how the RECLAIM program centered around wellness and how it might encourage veterans to pursue healthcare outside of illness. One veteran participant (V2) described RECLAIM as “*more like wellness than health care*” because it moves beyond the typical medical model (i.e., specific disease or symptoms) and considered the whole person.

“So having a program like this kind of gets past that first layer of oh, we’re just going to treat your symptom. [This program] is thinking past that. You’re not just treating a symptom. – Veteran (V11)

“If your body is not doing well, then your mind doesn’t do well. And a lot of doctors, they’ll just throw some meds at mental health to try to make you feel better and they don’t take into consideration the exercise, the eating, the environment that you’re in, or the social aspects.” – Veteran (V14)

##### Proactive care

Several stakeholder participants discussed how RECLAIM was “*preemptive*” (VA clinician, also a veteran; S1) and “*[liking] the idea of being proactive instead of reactive*” (YMCA stakeholder). A VA clinician (S5) also expressed how programs like RECLAIM are “*a good way to start that paradigm shift for more preventative help*.”

“The VA’s system is kind of set to react. And this is the first time we are shifting our model to prevention, which I think it’s a good time to do it with the [younger veterans]. So I think [RECLAIM] is a neat way to get it started.” – VA clinician (S10)

Several participants suggested that veterans may engage with RECLAIM and acquire information that encourages them to seek additional care and may positively impact their health long-term. As such, the RECLAIM program was considered a valuable opportunity to help connect veterans to their VA benefits.

“I think you would have to say it’s something connected with the VA, because I don’t think a lot of people, a lot of soldiers, don’t know how to get into the VA. …But I think saying something like this is VA, like associated with the hospital would be big because then veterans are going [to understand] like this is the information you need for whatever [benefits]. – Veteran (V1)

Some participants expressed how the mindfulness component of the RECLAIM program could help veterans 1) be more attuned to what was going on with their health, 2) understand why they feel the way they do, and 3) recognize that “*[they’re] in control of all of these things”* (Veteran, V13).

“You know mindfulness is huge for everybody. But being self-aware is, sometimes we don’t realize our problems until we see ourselves more clearly. And I think that that’s a really great benefit to the program if they can be more mindful of – oh I didn’t realize I was feeling this way, or I didn’t realize how my behaviors or habits were shaping my mind state or you know my health. – VA clinician (S8)

“Like it’s just the world that we live in, how busy we are, and how much we put on our plates that we…. It’s very easy to be unmindful, which makes us feel more disconnected.” – Veteran (V9)

##### Community-based setting

Participants indicated that the space for conducting the program “*feels less clinical*” (Veteran, V2) than a hospital setting and would provide a “*quiet space*” (Veteran, V3) for veterans to focus on things like meditation and breathing.

“So I think if we you know do more community partners and take things out of the stigma of the VA … I think getting out into the clinic is a wonderful way to think outside the box.” – VA clinician (S10)

“I mean I love my country but flags and military logos and the president and all that I mean it’s not going to feel like a relaxing environment to me, you know? …if you really want me to relax, that’s probably not the best way.” – Veteran (V2)

Some veteran participants also discussed how the environment of a VA medical center may cause unpleasant feelings and that for some veterans “*it’s very stressful to go to the VA*” and “*it is depressing [and] very sad*” (Veteran, V1). Another veteran (V4) didn’t think the setting would make a drastic difference for most veterans and some veterans may positively view the VA environment “*because everybody’s there*.”

#### Using health promotion programs to help veterans establish structure in their daily lives

Veterans discussed how they had grown accustomed to the structure of the military and “*how everything was provided*” and that “*when [veterans] get out, [they] go through a loss period and [they’re] trying to find a connection*.”

**“**I’m out and I’ve never done anything else. I don’t know how to be an adult. Because the military told me what to wear, when to be there. They provided me a nice check…gave me clothes, food, and everything. So for me to get out, I have to do it all by myself, everything.” – Veteran (V8)

“I know that a lot of military members struggle to assimilate to civilian life because they have such a structured lifestyle while they’re in the military that when they’re out on their own, they have nobody to help hold them accountable besides themselves.” – YMCA stakeholder (S24)

Participants agreed that providing structure would be especially beneficial for younger veterans who entered the service prior to acquiring a sense of independence and “*don’t have that experience and don’t know the questions to ask”* (VA clinician, S13) when they get out of the military.

“[The military] is a high stress job. But then even when you get out it’s different because now, you’re [experiencing] a very different stress. The stress they’re used to is military stress. That is something you can handle because you’re going to do it every day.” – Veteran (V10)

##### Establishing a mission/purpose

Participants expressed that it was important for veterans to have a sense of purpose and passion because, if they “*don’t have purpose in life, [they’re] not going to be driven to take care of yourself because there’s nothing that you’re striving for*” (VA clinician, S14).

“It’s easy for people to push past some of these barriers and start reclaiming their life if they have that mission or purpose.” – YMCA Stakeholder (S26)

Veterans discussed how “*you know what’s expected of you*” (Veteran, V1) in the service and this creates a “*very strong purpose, drive, [and] mission*” (Veteran, V12). However, this purpose, drive, and mission becomes lost in translation when veterans exit the service. Participants expressed how RECLAIM could help veterans establish a “*new normal*” (VA clinician, S6) within the civilian world, while also acquiring additional skills and resources.

“If you’re out of the military and that was your mission, like taking some time over those 8 weeks to maybe develop what that is and then reinforce it with health care skills that help them to build off of it.” – VA clinician (S4)

“If you don’t have a sense of purpose, like you’re starting to fall into some [stuff], you know, this [program] is a tool that you can use if you start feeling that you’re falling by the wayside.” – Veteran (V14)

##### Building a support system

Participants discussed how RECLAIM could help veterans rebuild a support system. Social support was considered an important aspect of the program because there is a tendency to “*isolate yourself when you get out*,” which “*creates so many downstream effects*” (VA clinician, also a veteran; S1).

“You had that brother and sisterhood [in the service] and I think that [RECLAIM] would be a great thing to have, especially to include into tools for new veterans.” – Veteran (V13)

One veteran (V11) indicated he “*didn’t think there was much of a veteran community around*.” Participants expressed that the RECLAIM program could help strengthen the veteran community by actively connecting veterans and their families with community resources. Some stakeholders echoed these considerations about helping veterans “*understanding the resources that are available to them*” (VA clinician, S14). Stakeholders also viewed RECLAIM and its positioning within a community-based organization as an “*opportunity to create partnerships*” (VA clinician, S10) in the community.

Inclusion of family, especially spouses or significant others, was frequently discussed and suggested for consideration within RECLAIM to help veterans re-establish their support system. However, there was some inconsistency about whether inclusion of family was always a positive and necessary aspect of the program; one veteran (V2) shared that the program could help “*connect [veterans] with [family],”* while others thought some veterans may *“[not] want [family] knowing what [they’re] struggling with*” (YMCA stakeholder, also a veteran; S25).

### Theoretical analysis of emergent themes

As presented earlier, the Interpersonal Psychological Theory of Suicide (IPT) [5] and the Self-Determination Theory (SDT) [14, 15] guided the development of the RECLAIM program. The IPT is comprised of two constructs: thwarted belongingness and perceived burdensomeness. The SDT focuses three universal psychological needs: relatedness, competence, and autonomy. The section below discusses how the study’s emergent themes align with the constructs from both the IPT and the SDT. This alignment is also presented in Table 2.

#### Theme 1

Participants frequently discussed the importance of using familiar language when promoting RECLAIM and presenting program content. Additionally, peer-to-peer outreach (another way to create familiarity) was often suggested. These suggestions are consistent with the theoretical constructs of belonging (IPT) and relatedness (SDT); program materials and content familiar to veterans can provide them with the sense of being connected to the program and its content which, as participants indicated, can increase the likelihood of buy-in and continued engagement.

#### Theme 2

Participants were drawn to the idea that RECLAIM is proactive and focuses on aspects of the whole person to improve well-being. Some participants also expressed how the mindfulness component of RECLAIM will help veterans be more attuned to and understanding of their health needs and recognize that they are in control. These aspects support the needs of autonomy (SDT) and competence (SDT). The proposed mindfulness activities also aim to improve veterans’ resiliency and coping skills, allowing them to independently address difficult situations or emotions, and prevent the perception that they are a burden on others (IPT). Finally, some participants discussed the benefits of the community-based setting, which can improve veterans’ sense of belonging (IPT) and relatedness (SDT) by connecting them with other veterans in their community.

#### Theme 3

When veterans transition out of the military, the structure (e.g., financial support, day-to-day schedules) they had become accustomed to is not readily apparent when they leave the service. Therefore, participants perceived RECLAIM as an opportunity to assist veterans’ efforts to replicate the structure of the military into their civilian lives. Providing veterans with structure addresses all five theoretical constructs of both the IPT and the SDT. First, by establishing a new purpose or mission, and related goals, veterans will be able to feel more autonomous (SDT) and competent (SDT) and, therefore, can perceive themselves as less burdensome (IPT) to others. In addition, RECLAIM can facilitate veterans’ sense of belonging (IPT) and relatedness (SDT) by connecting them with other veterans who will be able to identify with their experiences, concerns, struggles, etc. The inclusion of family members, as suggested by some participants, can also increase veterans’ sense of belonging.

## Discussion

The RECLAIM health promotion program was designed to facilitate post-9/11 veterans’ social and self-connectedness to reduce the risk of engaging in suicidal behaviors. The purpose of this study was to elicit veteran and stakeholder feedback to provide an in-depth understanding of participants’ perceptions of RECLAIM, which would be considered for the program design and implementation. In addition to overall positive perceptions of RECLAIM, findings revealed three overarching themes: 1) enhancing program recruitment and retention, 2) the perceived ability of a health promotion program to provide more holistic, veteran-centered care, and 3) using health promotion programs to help veterans establish structure in their daily lives. While the findings from this study specifically inform the ongoing development and future implementation of RECLAIM, many of the principles discussed are relevant for the design and implementation of other programs focused on health and wellness for post-9/11 veterans, including those aimed at reducing the risk of suicidal behaviors in this population.

Veterans expressed concern over some of the proposed terminology presented in the focus group discussions. In particular, the proposed concept of a ‘lifestyle’ program was not generally well-received; participants indicated that term was “invasive,” “vague,” and created a perception that there was something wrong with veterans’ current lifestyles. Similarly, veterans in previous studies have indicated a general distrust with the VA healthcare system and add that vague language can fuel this distrust and is off-putting [23, 24]. Therefore, it is important to consider alternate terminology to eliminate this as a potential barrier to participating in the RECLAIM program. As such, the findings from this study regarding the use of the word lifestyle and the negative feelings it elicited among our participants forced us to consider alternate wording (i.e., holistic health promotion program) to provide a clear and more positive perception of RECLAIM to veterans. Providing clear explanations is consistent with recommendations from a previous study to reduce lack of understanding as a barrier to participation [25]. Additionally, being transparent with messaging and purpose has been suggested for trauma survivors, such as veterans [26]. Considering the possibility of having combat veterans who have experienced trauma as participants in RECLAIM, this recommendation is important to incorporate within promotional items and program content.

Mindfulness-based interventions are among the most commonly used complementary and integrated health (CIH) approaches within the VA [27]. However, participants in this study repeatedly emphasized the importance of providing clarity and a sense of familiarity when promoting RECLAIM. This indicates that even though the popularity of mindfulness-based interventions has increased, veterans may still have some uncertainty about these types of interventions. To clarify the purpose of RECLAIM and its potential benefits, participants in the current study recommended using peer-to-peer outreach (i.e., having former veteran participants promote RECLAIM) to promote the program. The use of peers has been suggested in other studies to generate veteran buy-in and trust [27–29]. The use of fellow veterans to promote health care or a specific program or treatment is a strategy that has been previously suggested considering that peers can be viewed as allies [27]. Using peer-to-peer outreach may also help overcome stigma associated with receiving care and can improve treatment entry and adherence. The ability of peers to increase veterans’ willingness to access health care services and programs can have positive, long-term effects can improve their overall health and wellness and, ultimately, help reduce their risk for engaging in harmful behaviors.

Several participants considered RECLAIM to be an opportunity to help veterans connect to and understand available services at the VA. Some veteran participants indicated being unaware of VA benefits prior to exiting the military and having limited knowledge regarding the processes to obtain benefits. This is critical to address considering VA services, such as nutrition and weight loss, can help facilitate proactive behaviors by helping veterans make healthy decisions that will positively impact their overall and long-term health. Similarly, these challenges were expressed by veterans in previous studies who reported difficulty navigating the VA system due to a lack of understanding or misunderstanding available services and benefits [24, 30]. Unfortunately, previous research has found that most veteran-directed programs do not include components to assist veterans with accessing their benefits [25]. Benefits that veterans may be eligible for can play an important role in long-term health and wellness. For instance, education benefits can enable veterans or their family members to achieve a higher level of education. Education is positively associated with many health and well-being outcomes (e.g., greater income) and is only one example of the benefits available to veterans. The difficulties associated with a lack of awareness regarding VA services and benefits are also important to address given that most veterans who have died by suicide had not been receiving VA care [6], which may stem from the challenges and confusion previously mentioned. Programs such as RECLAIM can play an important role in helping veterans connect to their health care benefits and, as such, it is important that program facilitators are knowledgeable in available services and steps to enrollment.

When individuals enter into military service, they adopt military norms, values, and language through basic training [31, 32]. While the military provides individuals with structure, a sense of purpose, and close social bonds, it forces detachment from previous social support systems, sense of self, and lives as civilians. The lack of structure and direction was addressed by participants in this study and, similarly, was addressed by veterans in a previous study who indicated that this type of structured environment provides clarity and simplicity to decisions and procedures [3]. The loss of a familiar, structured environment also impedes reintegrating veterans’ ability to organize their own life and find a sense of meaning and purpose without clearly specified goals. When veterans reintegrate back into the community, there is no assistance comparable to basic training to help them re-acclimate to being a civilian [33, 34], which can leave veterans feeling lost and disconnected. As presented by the participants in this study, health promotion programs can (and should) assist veterans with establishing goals that can generate structure and purpose outside of the military. This is especially important considering that structure and purpose have been identified as psychosocial protective characteristics against suicidal behaviors [35, 36].

### Limitations

Participants in this study have not actually participated in the RECLAIM program but were responding to and providing their perceptions of a description of the program. Perceptions of RECLAIM could change based on a veteran’s actual experience engaging in the program and its proposed activities. All veteran participants indicated they were receiving VA care at the time of study participation, which limits the understanding of how veterans not enrolled in VA care may perceive RECLAIM. Additionally, veteran and VA clinician participants in this study were from a single VA facility and perspectives may be limited based on the experiences at this specific location. Some focus group participants may not have felt comfortable sharing their opinions and, therefore, their perspective may not be represented in the findings. Finally, the overall positive response to the RECLAIM program may have been due to selection bias introduced in the convenience sampling method.

## Conclusions and future research

The purpose of this study was to elicit feedback from veterans and stakeholders regarding initial perceptions of the RECLAIM program and considerations for future implementation. Formative research in the RECLAIM program design protocol ensures the program is contextually appropriate to the needs of post-9/11 veterans. Discussions in this study were specifically directed at the RECLAIM program and the feedback acquired in this study will be considered in further refinement and future implementation of the RECLAIM program. However, the findings from this study can be considered for the development and implementation of other related programs designed to improve the health and wellness of post-9/11 veterans and/or reduce their risk for engaging in suicidal behaviors. Initial feedback from participants in this study indicate that RECLAIM is an acceptable and veteran-centric program. Future research may consider incorporating the aspects of RECLAIM that participants indicated were appealing into other programs for post-9/11 veterans. In addition, findings from this study also highlighted barriers and facilitators to program implementation, along with strategies for recruiting and retaining participants. These are barriers and facilitators that future work should consider and proactively address to increase veterans’ ability to successfully engage with veteran-directed programs and interventions. Finally, future research should continue to evaluate how the interpersonal psychological theory of suicide and the self-determination theory inform each other to enhance our understanding of how to effectively address and improve protective factors among veterans to reduce their risk of suicidal behaviors.

## Supplementary information

**Additional file 1.**

## Data Availability

The interview guide is provided as Supplementary file 1. Additional data are available upon request to the corresponding author (SS).

## Abbreviations

CIH: Complementary and integrated health
RECLAIM: REconnecting to Civilian Life using Activities that Improve Mindfulness
VA: Department of Veterans Affairs
YMCA: Young Men’s Christian Association
