# Supplementary material for: Reducing veterans’ risk for suicidal behaviors: a qualitative study to inform development of the RECLAIM health promotion program
Source: BMC Health Serv Res. 2020 Aug 1;20:707. doi: 10.1186/s12913-020-05587-7 (PMC7395384; doi:10.1186/s12913-020-05587-7)
Supplement: Supplementary file 1 — Additional file 1. [file 12913_2020_5587_MOESM1_ESM.docx]

Discussion questions to be asked after welcome and introduction, reviewing study information sheet (and surveys for veteran focus groups), the discussion guidelines, and the program description.

**Discussion Questions:**

1. What do you think of this program?
   1. Probe: What aspects of the program do you find most appealing? Least appealing?
2. What, if any, changes would you make to the RECLAIM program based on the description provided?
3. How can this program benefit veterans?
4. What do you think would keep veterans from participating in RECLAIM?
5. What do you think of the sample support materials?
6. *Question dependent on group type:*
   1. [Veterans] What about the RECLAIM program would make you want or not want to participate?
   2. [Stakeholders] What about the RECLAIM program would make you refer or recommend veterans to (or not) seek it out?
7. Have we missed anything?
8. Is there anything else that you want to share that we haven’t talked about yet?
